# Carboxyl-Terminal Truncated HBx Regulates a Distinct MicroRNA Transcription Program in Hepatocellular Carcinoma Development

**DOI:** 10.1371/journal.pone.0022888

**Published:** 2011-08-04

**Authors:** Wing-Kit Yip, Alfred Sze-Lok Cheng, Ranxu Zhu, Raymond Wai-Ming Lung, Daisy Pui-Fong Tsang, Suki Shuk-Kei Lau, Yangchao Chen, Jonathan Gabriel Sung, Paul Bo-San Lai, Enders Kai-On Ng, Jun Yu, Nathalie Wong, Ka-Fai To, Vincent Wai-Sun Wong, Joseph Jao-Yiu Sung, Henry Lik-Yuen Chan

**Affiliations:** 1 Institute of Digestive Disease, The Chinese University of Hong Kong, Hong Kong SAR, China; 2 Department of Medicine and Therapeutics, The Chinese University of Hong Kong, Hong Kong SAR, China; 3 Department of Anatomical and Cellular Pathology, The Chinese University of Hong Kong, Hong Kong SAR, China; 4 Department of Surgery, The Chinese University of Hong Kong, Hong Kong SAR, China; University of Hong Kong, Hong Kong

## Abstract

**Background:**

The biological pathways and functional properties by which misexpressed microRNAs (miRNAs) contribute to liver carcinogenesis have been intensively investigated. However, little is known about the upstream mechanisms that deregulate miRNA expressions in this process. In hepatocellular carcinoma (HCC), hepatitis B virus (HBV) X protein (HBx), a transcriptional *trans*-activator, is frequently expressed in truncated form without carboxyl-terminus but its role in miRNA expression and HCC development is unclear.

**Methods:**

Human non-tumorigenic hepatocytes were infected with lentivirus-expressing full-length and carboxyl-terminal truncated HBx (Ct-HBx) for cell growth assay and miRNA profiling. Chromatin immunoprecipitation microarray was performed to identify the miRNA promoters directly associated with HBx. Direct transcriptional control was verified by luciferase reporter assay. The differential miRNA expressions were further validated in a cohort of HBV-associated HCC tissues using real-time PCR.

**Results:**

Hepatocytes expressing Ct-HBx grew significantly faster than the full-length HBx counterparts. Ct-HBx decreased while full-length HBx increased the expression of a set of miRNAs with growth-suppressive functions. Interestingly, Ct-HBx bound to and inhibited the transcriptional activity of some of these miRNA promoters. Notably, some of the examined repressed-miRNAs (miR-26a, -29c, -146a and -190) were also significantly down-regulated in a subset of HCC tissues with carboxyl-terminal HBx truncation compared to their matching non-tumor tissues, highlighting the clinical relevance of our data.

**Conclusion:**

Our results suggest that Ct-HBx directly regulates miRNA transcription and in turn promotes hepatocellular proliferation, thus revealing a viral contribution of miRNA deregulation during hepatocarcinogenesis.

## Introduction

The hepatitis B virus (HBV) X protein (HBx), a 154-amino acid transcriptional *trans*-activator, is believed to play an oncogenic role in the development of hepatocellular carcinoma (HCC) [1]–[3]. Despite having no DNA binding domain, HBx can deregulate cellular gene expression by altering various signal transduction pathways [4]–[6], associating with transcription factors or components of basal transcription machinery [7], [8], and inducing epigenetic modifications [9]. HBx is frequently integrated into the host genome in truncated form without its carboxyl-terminus and over-expressed in HBV-associated HCC tissues [10]–[13]. These carboxyl-terminal truncated HBx (Ct-HBx) variants have been shown to abrogate the growth-suppressive and apoptotic effects of full-length HBx [10], [11], [14]. Moreover, Ct-HBx is able to enhance cellular proliferation *in vitro* [11], [14] and tumorigenicity *in vivo* [13], [15]. However, the underlying mechanisms remain to be fully defined.

Similar to HBV, human papillomavirus (HPV), Epstein-Barr virus (EBV) and human T-lymphotropic virus Type I (HTLV-1) express different oncoproteins which have been strongly associated with the development of cancers [16]–[18]. These oncoproteins not only control the transcription of protein-coding genes, but also microRNAs (miRNAs), which belongs to a family of small non-coding RNAs (∼18–22 nt) that regulates gene expression by either directing mRNA degradation or repressing post-transcriptional translation. It has become apparent that specific miRNAs contribute to neoplastic transformation and tumorigenesis through influencing the translation of multiple key cellular genes and thereof crucial biological processes. In this connection, HPV oncoprotein E6 has been shown to suppress miR-34a transcription [16] while LMP1 and Tax encoded by EBV and HTLV-1, respectively, have been demonstrated to up-regulate miR-146a that was found to be differentially expressed in cancers [17], [18]. Wang *et al.* have recently demonstrated the ability of HBx to modulate cellular miRNA expression in HCC cells [19]. However, the systemic effect of HBx in miRNA regulation in human hepatocytes remains unclear. More importantly, whether miRNAs could be transcriptionally controlled by Ct-HBx, the more preferentially-expressed form of HBx in HCC tissues has not been explored.

In this study, we tested the hypothesis that the frequent carboxyl-terminal truncated form of HBx contributes to liver carcinogenesis through deregulating cellular miRNAs. We directly compared the miRNA profiles in human hepatocytes expressing full-length HBx and Ct-HBx. We found that full-length HBx significantly decreased liver cell viability and increased the expression of a set of miRNAs with growth-suppressive functions. In contrast, Ct-HBx stimulated cellular proliferation which was concordant with the repression of the same subset of growth-suppressive miRNAs. Moreover, Ct-HBx was shown to bind to their promoter regions leading to transcriptional repression. Notably, some of these miRNAs were significantly down-regulated in a subset of HCC tissues with carboxyl-terminal HBx truncation compared to their matching non-tumor tissues, highlighting the clinical relevance of our data and the importance of Ct-HBx during hepatocarcinogenesis.

## Materials and Methods

### Patient samples and cell lines

Human tissue samples were collected with written consent of the patients and prior approval from Joint CUHK-NTEC Clinical Research Ethics Committee. Sixteen pairs of HCC and the matching non-tumor tissues were collected from HBV-associated patients between 2004 and 2006 at the Prince of Wales Hospital in Hong Kong (Table 1). Non-tumorigenic human liver cell lines: MIHA, immortalized from a HBV-negative patient with the SV40 T antigen [20] and LO2, immortalized by human telomerase reverse transcriptase over-expression [21] as well as human embryonic kidney (HEK) 293T cells and PLC5 HCC cells were cultured in Dulbecco's modified Eagle's medium (Invitrogen, Carlsbad, USA) supplemented with 10% Fetal Bovine Serum (Thermo Scientific HyClone, Logan, USA) and maintained at a 37°C humidified incubator with 5% CO_2_.

**Table 1:** Characteristics of the HBV-associated HCC patients and C-terminal truncation status of the corresponding HBx gene in tumor.

Case no.	Sex	Age	Cirrhosis	HBx truncation	*F1/R1		F1/R2		F1/R3		F1/R4		F1/R5	
					NT	T	NT	T	NT	T	NT	T	NT	T
508	F	46	Y	Y	+	+	+	+	+	+	+	+	+	−
513	F	39	Y	N	+	+	+	+	+	+	+	+	+	+
515	M	60	Y	Y	+	+	+	+	+	+	+	+	+	−
519	M	52	Y	Y	+	+	+	+	+	+	+	+	+	−
522	F	42	Y	N	+	+	+	+	+	+	+	+	+	+
527	M	59	Y	Y	+	+	+	+	+	+	+	+	+	−
531	M	68	N	Y	+	+	+	+	+	+	+	+	+	−
532	M	71	Y	N	+	+	+	+	+	+	+	+	+	+
547	M	59	N	N	+	+	+	+	+	+	+	+	+	+
550	M	67	N	N	+	+	+	+	+	+	+	+	+	+
551	M	65	Y	Y	+	+	+	+	+	+	+	+	+	−
552	M	58	Y	Y	+	+	+	+	+	+	+	−	+	−
554	F	62	Y	N	+	+	+	+	+	+	+	+	+	+
557	M	64	Y	N	+	+	+	+	+	+	+	+	+	+
558	F	55	Y	N	+	+	+	+	+	+	+	+	+	+
559	M	53	N	Y	+	+	+	+	+	−	+	−	+	−

*Primer pairs used for HBx amplification (see Figure 5A).

Abbreviations and symbols: NT, matched non-tumor tissue; T, tumor tissue; +, successful amplification of HBx fragment; −, unsuccessful amplification of HBx fragment.

### Cloning and detection of HBx gene by PCR

Viral DNA samples extracted from the sera of HCC patients CH230 and BC265 were used for the amplification and cloning of HBx gene as previously described [22]. Full-length HBx was also cloned using a pBR-HBadr4 plasmid template originated from HBV subtype *adr* [23]. Deletion of HBx gene in HCC and the matching non-tumor tissues was detected by PCR in a 25-µl reaction as previously described [13]. The PCR products were analyzed by electrophoresis on a 1.5% agarose gel and visualized by GelDoc XR system (Bio-Rad Laboratories, Inc., Hercules, CA, USA).

### Generation of lentiviruses

**Figure 1:**
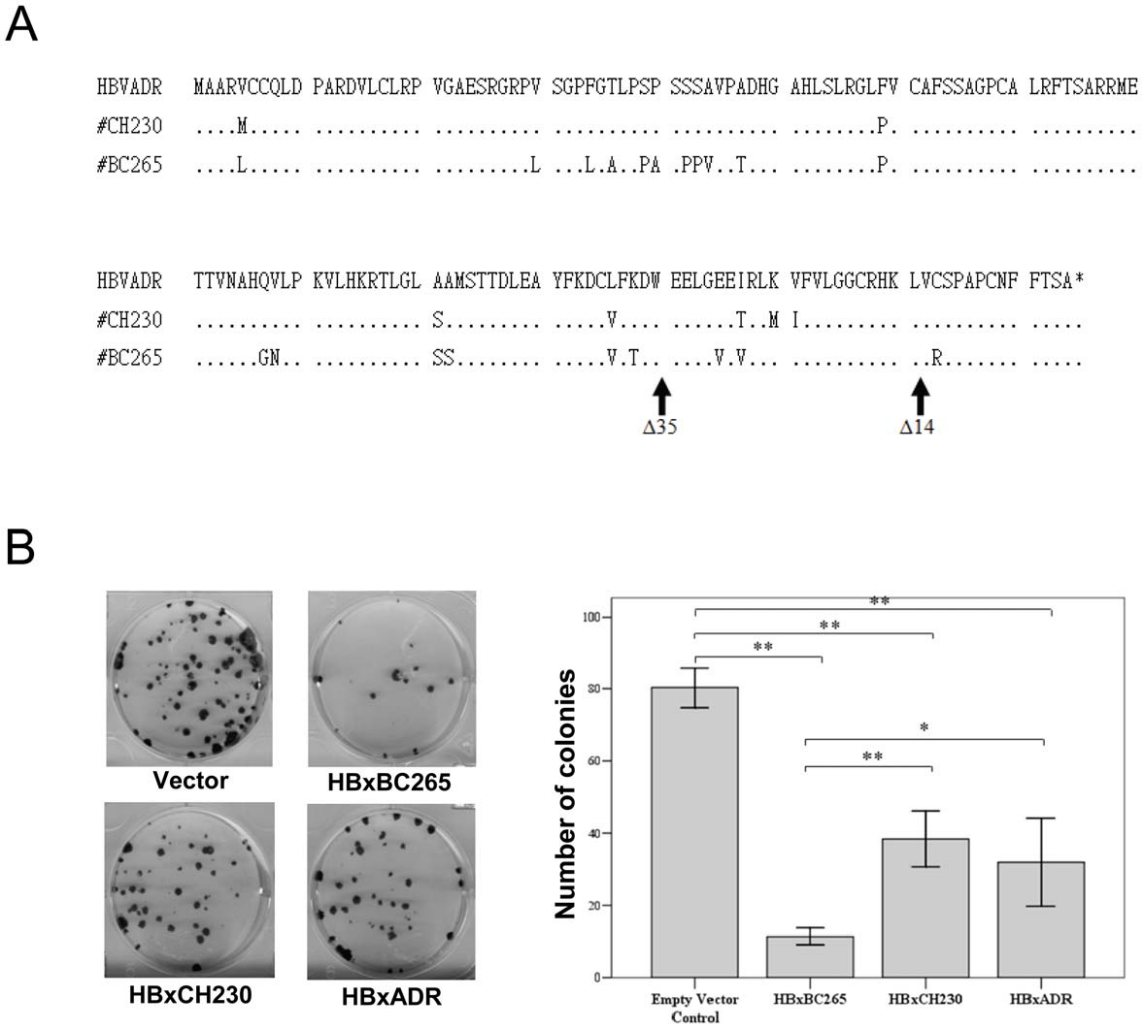
Selection of HBx gene from HBV-associated HCC patients. (A) Amino acid sequences of HBx gene derived from 2 HBV-associated HCC patients (CH230 and BC265). The sequence of HBV subtype *adr* is also shown at the top. Identical amino acid residues are represented by dots. The locations of truncation generated by PCR are shown in arrows. (B) Effect of different full-length HBx sequences on growth as assessed by the colony formation assay. Cells were transfected with plasmids expressing HBx derived from BC265, CH230, *adr* or vector alone. Results are derived from triplicate transfections (± SD). *, *p*<0.05; **, *p*<0.01.

**Figure 2:**
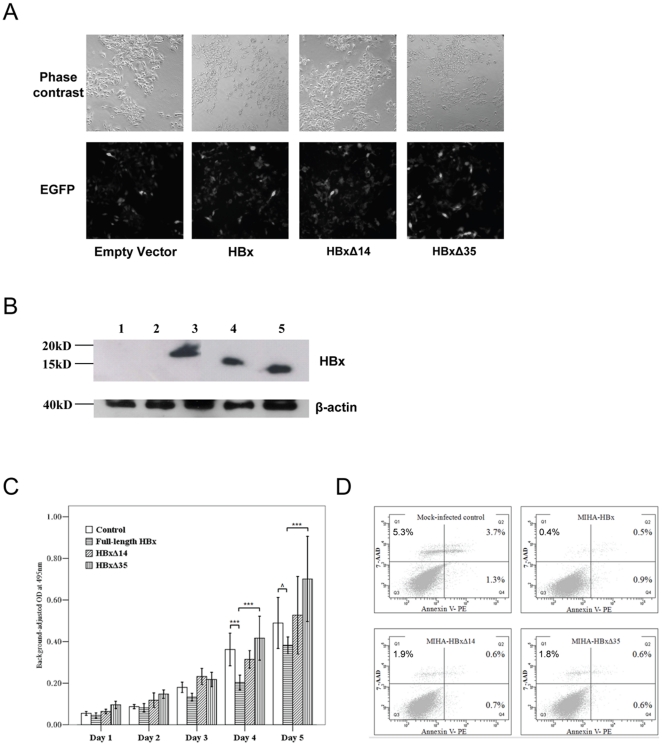
Expression and functions of full-length HBx and Ct-HBx in human hepatocytes. (A) Phase contrast and fluorescent microscopic examination of MIHA hepatocytes infected by lentivirus containing different forms of HBx or EGFP alone at day 3 post-infection. (B) Western blot analysis of HBx expression in lentivirus-infected MIHA hepatocytes. β-actin was used as loading control. 1) Parental MIHA, 2) EGFP only, 3) Full-length HBx, 4) HBxΔ14 and 5) HBxΔ35. (C) Effect of different forms of HBx on cell proliferation. Results are derived from 6 replicates of 2 independent experiments (± SD). ***, *p*<0.005; ∧, *p* = 0.06. (D) Effect of different forms of HBx on apoptosis as assessed by flow cytometry. Cells were stained with 7-AAD and Annexin V to determine cell viability and apoptosis, respectively.

HBx fragments from patient CH230 were amplified by PCR using a forward primer carrying a Kozak and flag-tag sequence and reverse primers with an artificial stop codon at different deletion sites. PCR products were cloned into a pCR2.1-TOPO vector (Invitrogen, Carlsbad, USA) and then sub-cloned to the *EcoR*I and *Sal*I restriction sites of a lentivirus vector, pRRL-cPPT-CMV-X-IRES-EGFP-PRE-SIN, to generate Lenti-X, Lenti-XΔ14 and Lenti-XΔ35 (expressing full-length, 14- and 35-amino acid carboxyl-terminal truncation, respectively, see Figure 1A). We chose to investigate HBxΔ14 and HBxΔ35 because these Ct-HBx have been shown to abrogate the growth-suppressive effects induced by full-length HBx, effectively promote cell transformation and enhance the proliferative activity of neoplastic cells [11], [14]. More importantly, they have been identified as natural deletion mutants in HCC tissues [11], [14]. Packaging of lentivirus was performed by transient transfection of HEK 293T cells with the transfer vector and packaging vectors, pMDL/pRRE, pRSV-REV and pCMV-VSVG as described previously [24]. Infection was carried out on 2×10^4^ of MIHA hepatocytes in a 24-well plate with 8 µg/ml polybrene (Aldrich Chemical Company Inc., Milwaukee, USA). The transduction efficiencies of the 3 constructs and EGFP vector control were 70% to 90% by fluorescence microscope at day 3 post-infection (see Figure 2A).

### Western blot analysis

Fifty micrograms of protein were resolved on a 15% SDS-PAGE. HBx antibody [X36C] (ab2741) (Abcam plc, Cambridge, UK) (1∶2000) and β-actin antibody (Santa Cruz Biotechnology, Inc., Delaware, USA) (1∶5000) were used for western blot analysis.

### MiRNA extraction and expression microarray

Total RNA containing small RNA was extracted using miRNeasy Mini Kit (Qiagen, Valencia, USA) according to the manufacturer's protocol. One hundred nanograms of total RNA labeled with Cy-3 fluorescent dyes were used for miRNA profiling using Agilent Human miRNA Microarray Kit (V2) (Agilent Technologies, Inc., Santa Clara, USA) that contains probes for 723 human and 76 viral miRNAs according to the manufacturer's protocol. The slide was scanned using an Agilent Microarray Scanner, and data was extracted using the Agilent Feature Extraction Software. GeneSpring software (Agilent Technologies, Inc., Santa Clara, USA) was used to analyze the microarray data after filtering the non-expressed probes and normalization by the 75% percentile shift method.

### Quantitative real-time PCR

The level of mature miRNAs was quantified by TaqMan MicroRNA Assays (Applied Biosystems, Foster City, USA). The quantitative PCR was carried out in a 10-µl reaction using 384-well plate by Applied Biosystems 7900HT Fast Real-Time PCR System. Data was normalized by RNU6B or 18S rRNA and the fold change was determined by the comparative cycle of threshold (Ct) method. All reactions were carried out in triplicate and blank controls were included in every reaction.

### Chromatin immunoprecipitation microarray (ChIP-chip) and ChIP-PCR

The ChIP assay was performed by using Transcription Factor Chromatin Immunoprecipitation Kit (Red ChIP Kit) (Diagenode, Liège, Belgium) according to the manufacturer's protocol. In brief, HBxΔ35-, full-length HBx- and EGFP-expressing MIHA hepatocytes were crosslinked in 1% formaldehyde for 5 minutes at 37°C and collected. The cells were sonicated to shear the cross-linked chromatin into an average DNA fragment size of 200–600 bp. Five micrograms of antibody against HBx antibody (X36C) were used for immunoprecipitation. After overnight incubation, DNA-protein-antibody complex was eluted. The crosslinks were reversed by heating the samples at 65°C for 4 hours. DNA was extracted by the phenol∶chloroform∶isoamyl alcohol, ethanol-precipitation and resuspended in 10 µl of water. DNA was amplified by GenomePlex® Complete Whole Genome Amplification (WGA) Kit (Sigma, St. Louis, MO, USA) according to the manufacturer's protocol. In brief, 10 µl of DNA was subjected into library preparation and the first round of amplification. After purification by QIAquick PCR Purification Kit (Qiagen, Valencia, USA), 100 ng of DNA was subjected into the second round of amplification. Incorporation of aminoallyl-dUTP into 2 µg ChIP-DNA was done by using a random primed Klenow polymerase reaction (Invitrogen, Carlsbad, USA) at 37°C for 3 hours. Cy5 and Cy3 fluorescent dyes (GE Healthcare, Little Chalfont, UK) were coupled to MIHA-HBxΔ35 and MIHA-EGFP ChIP-DNA, respectively. The labeled samples were hybridized with Agilent Human Promoter ChIP-on-chip Microarray set at 65°C for 40 hours. Replicate dye-swap experiments were performed. After washing the arrays according to the manufacturer's protocol, arrays were scanned on GenePix 4000B scanner and data were extracted by using Agilent Feature Extraction Software (Agilent Technologies, Inc., Santa Clara, USA). For validation, quantitative real-time ChIP-PCR was performed by SYBR Green (Applied Biosystems, Foster City, USA) using the 7500 Real-time PCR System (Applied Biosystems, Foster City, USA) as previously described [25].

### Luciferase reporter assay

The *Renilla* luciferase reporter control plasmid, pRL-CMV and the pGL3 promoter luciferase reporter plasmid were purchased from Promega Corp (Madison, WI, USA). The reporter plasmids were generated by cloning the *Mlu*I-*Bgl*II DNA fragments amplified with specific primers targeting the upstream promoter regions of miR-26a (forward primer: 5′-ATATACGCGTGGATGCTTCATCATCCTCC-3′; reverse primer: 5′-ATATAGATCTCGGGAGAGAATTTTGACC-3′) and miR-29c (forward primer: 5′-ATATACGCGTCTAGAGGCCTGAAAGGAAG-3′; reverse primer: 5′-ATATAGATCTGACTGATGGTGTCGATGTG-3′). To analyze the miRNA promoter activity, 293FT cells (1×10^5^) grown in 24-well plates were co-transfected with 800 ng of expression vector (full-length HBx, HBxΔ35 or control plasmid) and 50 ng of reporter plasmid using Genejuice transfection reagent (Novagen, Madison, WI, USA). At 48 hours after transfection, cells were harvested and assayed using Dual Luciferase Reporter Kit (Promega, Madison, WI, USA) as previously described [26].

### Transfection of miRNA mimic

Hsa-miR-26a and negative control miRNA mimics were purchased from Ambion (Austin, TX, USA). Fifty nanomolar of miRNA mimics were transfected into PLC5 cells by using Lipofectamine 2000 (Invitrogen) according to the manufacturer's protocol. Protein and RNA were extracted 48 hours after transfection. Ectopic miRNA expression was confirmed by quantitative PCR using miScript Reverse Transcription kit and miScript SYBR Green PCR Kit (Qiagen, Hilden, Germany) as previously described [26]. The in-house designed miRNA specific primer sequences for miR-26a and snU6 are 5′-TTCAAGTAATCCAGGATAGGCT-3′ and 5′-ACGCAAATTCGTGAAGCGTT-3′ respectively.

### Colony formation assay

Full-length HBx fragments from HCC patients BC265 and CH230 as well as HBV subtype *adr* shown to induce HCC in transgenic model [27] were cloned into pcDNA™3.1/V5-His TOPO (Invitrogen, Carlsbad, USA). Two micrograms of plasmid DNA were transfected to 4×10^5^ cells on a 6-well plate using FuGENE6 Transfection Reagent (Roche Diagnostic Corp., Indianapolis, USA). Transfected cells were selected by 1000 µg/ml Geneticin G418 (Invitrogen, Carlsbad, USA). Fresh medium with G418 was replaced twice a week for 18 days. The colonies were visualized by crystal violet in methanol (0.5% w/v).

### Cell proliferation assay

Cell viability was assessed by a colorimetric method using CellTiter 96® AQ_ueous_ One Solution Cell Proliferation Assay (Promega Corp., Madison, USA) for 5 consecutive days. Two thousand cells were seeded to 96-well plate in 6 replicates. The plate was light-protected and assayed at 37°C for an hour. The absorbance of the colorimetric products formed was measured at 490 nm by a spectrophotometer.

### Apoptosis assay

Cell apoptosis was assessed by flow cytometry using a PE Annexin V Apoptosis Detection Kit I (BD Pharmingen, San Diego, USA) and analyzed by BD FACSAria™ Flow Cytometer and Modfit LT 3.0 (Verity Software House, Topsham, USA).

### Statistical Analysis

Statistical analysis was performed by GraphPad Prism (GraphPad Software Inc., San Diego, USA). The data was analysed by Student's t-test or Wilcoxon signed rank test. *P* value<0.05 was considered as statistically significant.

## Results

### Selection of HBx gene from HBV-associated HCC patients

To investigate whether full-length HBx and Ct-HBx regulate miRNA expression in human hepatocytes, we first selected HBx gene from HBV-associated HCC patients. We cloned the full-length HBx sequences from serum samples of 2 HCC patients and a reference control of HBV subtype *adr* [27]. Sequencing analysis verified that both the *adr* subtype and the patient CH230 sequences belonged to genotype C HBV while those of patient BC265 was genotype B (Figure 1A) and colony formation assay was then performed. As shown in Figure 1B, expression of all full-length HBx sequences in MIHA cells resulted in fewer number of colonies compared to cells transfected with vector only and the difference was statistical significant (*p*<0.01). This finding was consistent with previous study demonstrating the growth-inhibitory function of full-length HBx [11]. Among the full-length HBx sequences, we found that their colony formation abilities were different as MIHA expressing HBx derived from patient CH230 and *adr* formed significantly more colonies than that of patient BC265 (*p*<0.05, Figure 1B). These data were in agreement with our previous findings that patients infected by genotype C HBV had a higher incidence of HCC as compared to those infected by genotype B HBV [28], [29]. Thus, we selected the HBx gene derived from patient CH230 for the subsequent experiments.

### Effects of full-length HBx and Ct-HBx on cell growth and apoptosis

We then cloned the full-length and Ct-HBx genes into lentiviral vector and transduced the constructs into MIHA hepatocytes with high efficiency (Figure 2A). Over-expression of HBx genes was further confirmed by Western blot analysis (Figure 2B). Consistent with the finding of colony formation assay, MIHA hepatocytes expressing full-length HBx grew slower than vector control cells (*p*<0.005 and  = 0.06 on day 4 and 5, respectively, Figure 2C). On the other hand, the growth rates of MIHA expressing HBxΔ14 and EGFP vector were similar (Figure 2C), indicating that deletion of 14-amino acid from C-terminus lost the growth-suppressive effect of full-length HBx. Notably, the growth of MIHA expressing HBxΔ35 was consistently faster than the full-length counterpart (*p*<0.005 on both day 4 and 5, Figure 2C). To rule out possible cell line-specific effects, we determined the growth of an independent human hepatocyte cell line, LO2 expressing full-length HBx and HBxΔ35, and observed similar divergent effect on cell growth (Figure S1A).

Annexin V apoptosis assay was performed to examine the apoptotic effect of full-length HBx, HBxΔ14 and HBxΔ35. The early apoptotic and necrotic/late apoptotic cells were depicted in Quadrant 4 and 1/2, respectively, in Figure 2D. While the effect of different HBx forms on apoptosis appeared minimal in our system (Quadrant 4: HBx vs. control: 0.6–0.9% vs. 1.3%), all HBx forms decreased MIHA cell death (full-length HBx, HBxΔ14 and HBxΔ35: 1.8, 3.2 and 3%, respectively) compared to the vector control (10.3%). Collectively, our data confirmed with others that Ct-HBx abrogated the growth-suppressive function of full-length HBx which showed diverse effect on cell cycle distribution [10], [14].

### Deregulation of miRNAs by full-length HBx and Ct-HBx

**Figure 3:**
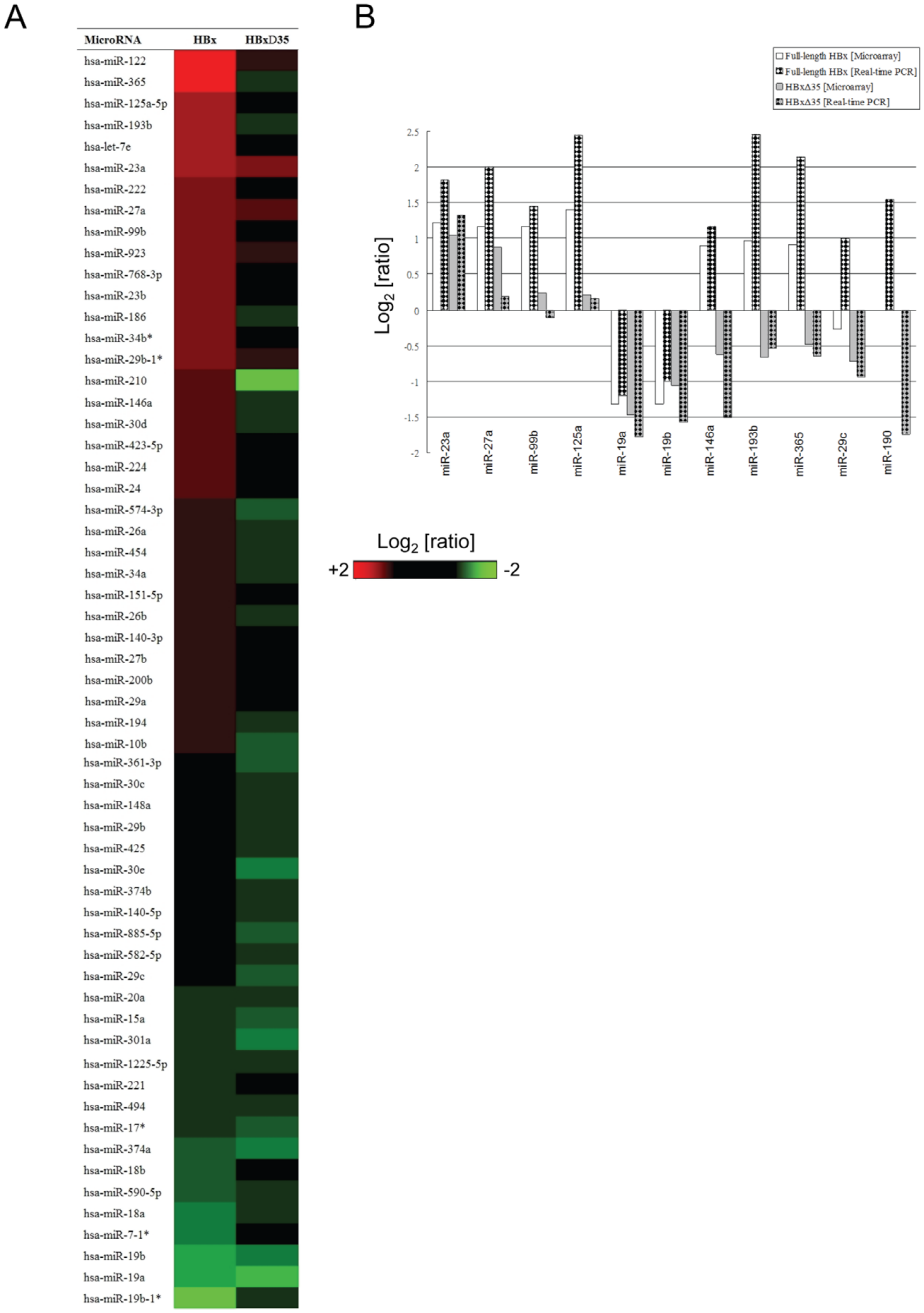
Differential miRNA expression by full-length HBx and Ct-HBx in human hepatocytes. (A) Heatmap of the differentially-expressed miRNAs by full-length HBx- and HBxΔ35-expressing MIHA hepatocytes compared to EGFP-expressing control cells. MiRNAs with at least 1.5-fold difference were identified using an expression microarray that contains probes of 723 human miRNAs. (B) Confirmation of the differentially-expressed miRNAs by real-time PCR analysis. Six and 5 miRNAs with common (left) and divergent (right) expression patterns, respectively, between full-length HBx and HBxΔ35 are shown. The miRNA expression was normalized with RNU6B or 18S rRNA and compared to EGFP-expressing control cells. Data are presented in Log_2_ scale.

We then performed miRNA microarray profiling to identify the potential miRNAs deregulated by full-length HBx and HBxΔ35 in MIHA hepatocytes. We hypothesized that full-length HBx and HBxΔ35 regulate distinct miRNA profiles based on their divergent effects on MIHA cell growth. Fifty-nine miRNAs were differentially expressed by full-length HBx and/or HBxΔ35 for at least 1.5-fold when compared to the vector control cells (Figure 3A). Full-length HBx activated and repressed a similar number of miRNAs in MIHA hepatocytes (Figure 3A). In contrast, the majority of the differential miRNAs was down-regulated by HBxΔ35 (Figure 3A). Only a few miRNAs were concordantly regulated by both HBx forms e.g. miR-23a up-regulation and miR-19a/b down-regulation (Figure 3A). Indeed, most of the miRNAs were divergently regulated by the two HBx forms. More than 80% (28/33) of the miRNAs up-regulated by full-length HBx were either down-regulated or unaffected by HBxΔ35 e.g. miR-146a, -193b, -210 and -26a/b (Figure 3A). On the other hand, two-third (24/36) of the miRNAs down-regulated by HBxΔ35 were either up-regulated or unaffected by full-length HBx e.g. miR-29b/c, -30d, -365 and -574-3p (Figure 3A). These data suggested that full-length HBx and Ct-HBx distinctively regulated the expression of a subset of miRNAs in human hepatocytes.

To validate the microarray findings, we performed TaqMan-based real-time PCR on 10 miRNAs showing common or divergent expression patterns between full-length HBx and HBxΔ35. Nine out of 10 expression patterns revealed by microarray could be confirmed by PCR analysis (Figure 3B). For example, miR-23a and -125a up-regulation as well as miR-19a/b down-regulation by both HBx forms were verified (Figure 3B). More importantly, the divergent regulation by full-length HBx and HBxΔ35 on miR-146a, -193b, -29c and -365 could be confirmed (Figure 3B). In addition, miR-190 not detectable by microarray was shown to be differentially regulated by full-length HBx and HBxΔ35 (Figure 3B), presumably due to superior sensitivity of real-time PCR. Altogether the PCR analysis confirmed the validity of array findings.

Overall, ten miRNAs were shown to be divergently regulated by the 2 HBx forms i.e. up-regulated by full-length HBx but down-regulated by HBxΔ35 (Table 2). Remarkably, eight out of the 10 miRNAs have been reported to exhibit anti-proliferative and/or pro-apoptotic functions in various cancer types including HCC (Table 2). We further confirmed the growth-inhibitory effect of miR-146a and -29 in MIHA and HepG2 liver cancer cells by cell proliferation assays (data not shown). Collectively, our findings suggested that the distinct repression of growth-inhibitory miRNAs by Ct-HBx may at least partially explain its growth-stimulatory effects on hepatocytes.

**Table 2:** A list of miRNAs divergently regulated by full-length and carboxyl-terminal truncated HBx.

	Fold Change		
MicroRNA	HBx/Vector	HBxΔ35/Vector	*Reference
Hsa-miR-26a	1.63	0.67	[30], [31]
Hsa-miR-26b	1.55	0.62	[32], [33]
Hsa-miR-29c	1.95	0.53	[34], [35]
Hsa-miR-30d	1.86	0.64	[36], [37]
Hsa-miR-146a	2.24	0.35	[17], [38]
Hsa-miR-190	2.91	0.31	-
Hsa-miR-193b	5.48	0.69	[39], [40]
Hsa-miR-210	1.98	0.25	[41], [42]
Hsa-miR-365	4.38	0.64	[43]
Hsa-miR-574-3p	1.63	0.56	-

*The references depicting the growth-inhibitory effects of miRNAs.

### Ct-HBx physically associates with miRNA promoters

**Figure 4:**
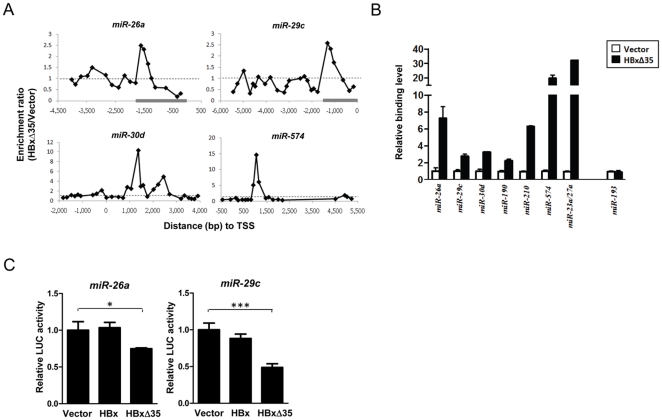
Direct transcriptional repression of miRNAs by Ct-HBx. (A) ChIP assays were performed with specific HBx antibody in HBxΔ35-expressing MIHA hepatocytes and EGFP-expressing (vector) control cells. ChIP-chip was performed to identify the promoters directly associated with Ct-HBx using a human promoter microarray that contains 17,861 protein-coding and 238 miRNA genes. The Y-axis of the HBx binding maps represents the enrichment ratio (HBxΔ35/Vector) while the X-axis represents the probe locations relative to the transcription start site (TSS) of miRNAs. Dotted lines indicative of no enrichment are shown as reference. Gray bars indicate the miRNA promoter regions cloned for luciferase assay. (B) Confirmation of Ct-HBx binding on the miRNA promoters by real-time PCR analysis. The immunoprecipitated DNA corresponding to the miRNA promoters was measured by quantitative PCR as a percent of input DNA. Results are relative binding level of HBx in HBxΔ35 compared to vector control cells. Data are presented in triplicates (± SD). miR-193 which shows no promoter binding in microarray serves as negative control. (C) Effect of Ct-HBx and full-length HBx on miRNA promoter activity in 293FT cells. Luciferase activity relative to *Renilla* control was measured. Data are presented in triplicates (± SD). *, *p*<0.05; ***, *p*<0.005.

We next investigated how Ct-HBx might regulate miRNA transcription. ChIP-chip analysis was performed to determine whether the promoter regions of the differentially-expressed miRNAs were bound by HBxΔ35 in MIHA hepatocytes. Interestingly, strong enrichment of HBx was detected in 6 HBxΔ35-down-regulated miRNAs (miR-26a, -29c, -30d, -190, -210 and -574) proximal to their transcription start sites (±1.5 kilo-base) in HBxΔ35-expressing MIHA hepatocytes compared with vector control (Figure 4A and data not shown). To validate the microarray findings, we performed real-time ChIP-PCR using primers specific to the bound promoter regions and observed significant enrichment by HBx antibody in HBxΔ35-expressing MIHA hepatocytes compared to vector control in all of the miRNA promoters (*p* = 0.0016 to 5.18E-05, Figure 4B). In addition to the repressed promoters, enrichment of HBx was also detected in the activated miRNA promoters. For instance, HBx binding was shown in the 4–4.5 kilo-base upstream promoter region of the miR-23a/27a cluster (Figure 4B) which was significantly up-regulated by HBxΔ35 (Figure 3). Overall, these data suggested that Ct-HBx could bind to the miRNA promoter regions for direct transcriptional regulation.

To compare the miRNA promoter binding patterns between Ct-HBx and full-length HBx, we also performed ChIP-chip in full-length HBx-expressing MIHA hepatocytes. In some of the direct HBxΔ35-repressed miRNAs, we observed different binding locations e.g. miR-29c promoter or no obvious binding e.g. miR-30d promoter by full-length HBx compared to HBxΔ35 (Figure S2). On the other hand, in the few miRNAs that were concordantly regulated by both full-length HBx and HBxΔ35 e.g. miR-23a and -27a, similar promoter region (around 4-kb upstream of TSS) was occupied by both forms of HBx (Figure S2). Real-time ChIP-PCR further validated the miRNA binding patterns in full-length HBx- and HBxΔ35-expressing MIHA hepatocytes (Figure S2). Taken together, these data demonstrated that full-length HBx also bound to some miRNA promoters, in either similar or different chromatin regions occupied by Ct-HBx.

### Ct-HBx directly represses miRNA transcription

To establish a direct link between Ct-HBx and transcriptional control of miRNA expression, we cloned the promoter regions of 2 repressed miRNAs (miR-26a and miR-29c) that contained the Ct-HBx binding sites (Figure 4A) into luciferase reporter and then co-transfected with HBxΔ35, full-length HBx or empty vector for promoter activity assays. As shown in Figure 4C, we found that both miR-26a and miR-29c promoter activities were significantly reduced by HBxΔ35 when compared to empty vector control (*p*<0.05 and 0.005, respectively). In contrast, there was no significant difference in the promoter activities between the full-length HBx-transfected and control cells (Figure 4C). Altogether, these data suggested that the truncated HBx, rather than the full-length counterpart, directly repressed transcription via physical binding resulting in decreased expression of these miRNAs.

### Expression of miRNAs in HCC tissues with preferential C-terminal HBx truncation

**Figure 5:**
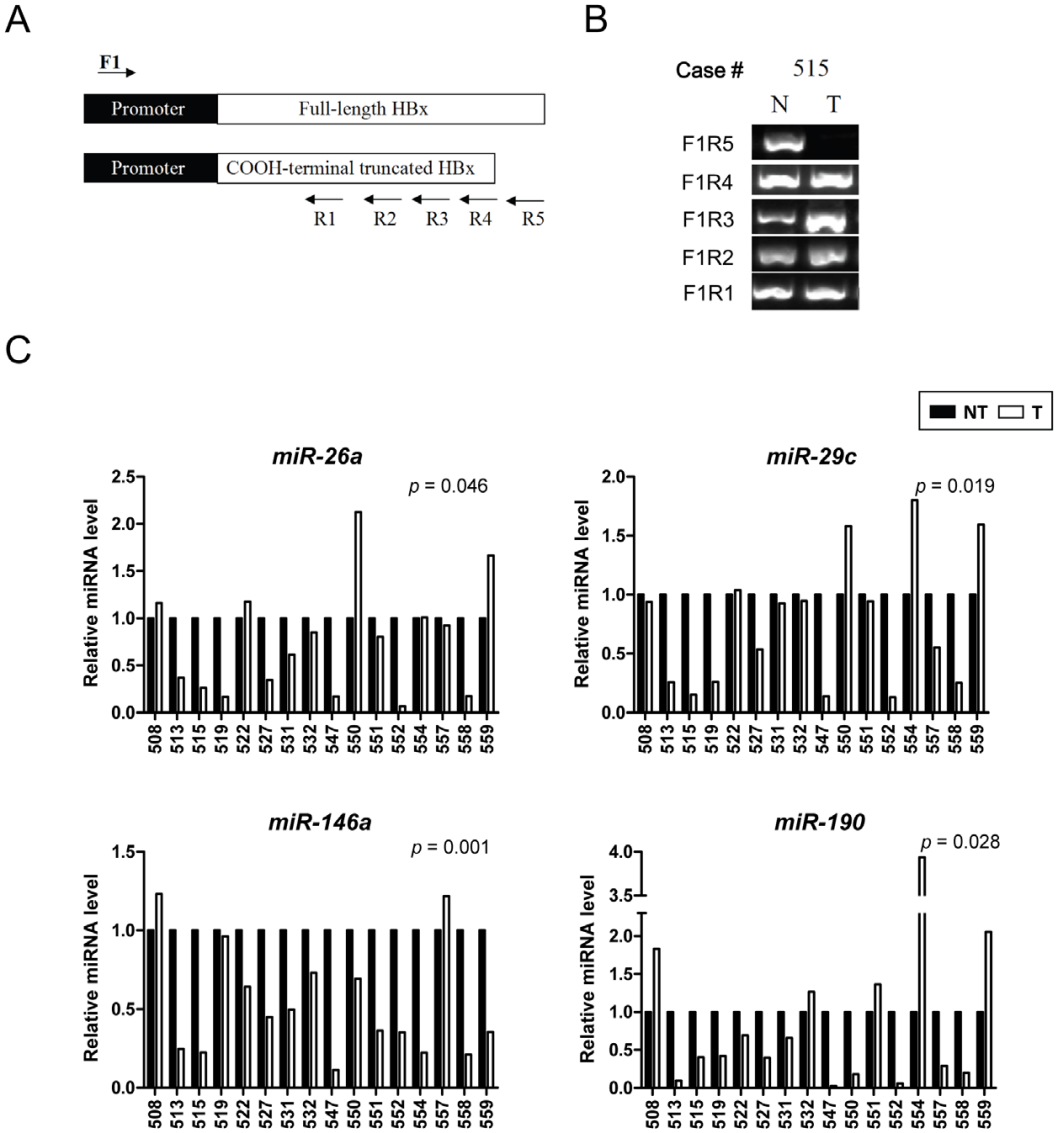
Expression of Ct-HBx-repressed miRNAs in HBV-associated HCCs and the matching non-tumor tissues. (A) PCR analysis of C-terminal HBx truncation in tissue specimens. A schematic diagram showing the positions of forward and reverse primers encompassing the promoter (F1) and C-terminus (R1 to R5) of HBx gene, respectively. (B) Detection of full-length HBx and Ct-HBx by PCR using different primer sets. Equal quantity of DNA was used for each PCR reaction as determined by GAPDH gene amplification (data not shown). NT, matching non-tumor tissue; T, HCC tumor tissue. (C) Expression of miRNAs in clinical specimens by real-time PCR analysis. The miRNA expression was normalized with RNU6B and the relative expression level between HCC tumors (T) and the matching non-tumor (NT) tissues are shown.

To investigate the clinical relevance of specific miRNA down-regulation by Ct-HBx, we examined the miRNAs expression in a cohort of 16 HBV-associated HCC and their matching non-tumor tissues. We first determined the HBx form (full-length or truncation) in the tissues by PCR analysis using 5 pairs of primers encompassing the entire and different lengths of the HBx gene (Figure 5A). An example of carboxyl-terminal deletion of HBx gene in a patient's tumor but not in the non-tumor tissue was shown (Figure 5B). Overall, full-length HBx could be amplified from all of the non-tumor tissues (16/16) but only in half of the HCC tissues (8/16) (Table 1). Thus, Ct-HBx was preferentially present in HCC tissues (*p* = 0.0024). Since both full-length and truncated HBx can be simultaneously present in the same HCC tissues [44], this PCR analysis might underestimate the number of tissue specimens harboring truncated HBx. Nevertheless, the high prevalence of Ct-HBx exclusively in HCC tissues confirmed its importance in human hepatocarcinogenesis.

We then examined the expression levels of some of the HBxΔ35-repressed miRNAs (miR-26a, miR-29c, -146a and -190) in the clinical specimens using TaqMan-based real-time PCR. Of the 8 HCCs exhibiting HBx truncation (Table 1), seven cases showed down-regulation of at least one of the examined miRNAs (Figure 5C). The levels of the examined miRNAs in HCC tissues were significantly lower than that of the matching non-tumor tissues (*p* = 0.001 to 0.046, Figure 5C). Overall, the 4 miRNAs were down-regulated by more than 2-fold in 43.8–62.5% of HCC tissues, an observation concurring with the notion that these miRNAs are at least partially repressed by Ct-HBx in HCCs.

## Discussion

Recent findings have shown that miRNAs are often deregulated in HCC and significantly correlated with its clinicopathological features, such as cirrhosis, metastasis, recurrence, and prognosis [45]–[47]. Functional studies further illustrate that miRNAs play important roles during hepatocarcinogenesis by directly influencing cell proliferation, apoptosis and metastasis of HCC cells [47], [48]. We and others have also delineated the miRNAs associated with HCC cells that were derived from chronic carriers of HBV and HCV, suggesting that miRNAs could be important mediators of HBV and HCV infection leading disease progression to HCC [49], [50]. However, the upstream mechanisms that contribute to the miRNA misexpression in HCC remain elusive. Here we provide evidence for miRNA deregulation in HCC through the HBV-encoded oncoprotein HBx. Using lentiviral-mediated expression system and integrative miRNA analysis, we found that full-length HBx and Ct-HBx distinctively regulated miRNA transcription in human hepatocytes. While full-length HBx activated and repressed similar numbers of miRNAs, its truncated form preferentially down-regulated miRNAs. Notably, at least 10 miRNAs were divergently regulated i.e. up-regulated by full-length HBx but down-regulated by the truncated counterpart. Our initial expression analysis in clinical samples implies that Ct-HBx at least partially contributes to the down-regulation of these miRNAs in human HCC, although further studies with larger sample size will be required to verify this notion.

Wang *et al.* were first to systematically demonstrate the direct role of full-length HBx in deregulating miRNAs and explore the downstream mechanisms leading to hepatocarcinogenesis [19]. In this study, seven and 11 miRNAs were found to be significantly up-regulated and down-regulated, respectively, in HBx-expressing HepG2 liver cancer cells compared to the control cells [19]. Albeit using different culture and expression systems, our findings also indicated that the same subset of miRNAs (miR-125a, -193b and -99b) were up-regulated by full-length HBx in human hepatocytes and thus supported the authenticity of data. Furthermore, we found that a substantial number of full-length HBx-induced miRNAs have been reported to possess growth-inhibitory functions (Table 2). For example, miR-193b, frequently down-regulated in HCC [40], melanoma [51], breast [52] and prostate cancers [53], was shown to promote apoptosis and inhibit growth of cancer cells [39], [40], [51]–[53]. Concurred with the apoptosis-enhancing property of HBx [19], [54], [55], these findings suggested that miRNAs could be mediators of full-length HBx-triggered growth-suppression.

In sharp contrast, we demonstrated that Ct-HBx distinctively repressed a subset of growth-inhibitory miRNAs that were induced by its full-length counterpart (Figure 3 and Table 2). These findings provide mechanistic insight into how Ct-HBx abrogates full-length HBx-induced apoptosis [10], [11] and stimulates hepatocellular growth [13]–[15]. Although HBx does not bind to DNA directly, it is capable to *trans*-activate transcription elements in the nucleus [4]. Recent studies have also shown that HBx achieves transcriptional suppression of important cancer-related genes e.g. TERT by enhancing promoter binding of transcription repressor like MAZ or physically interacting with the chromatin-modifying enzyme histone deacetylase 1 [56], [57]. In the present study, we show for the first time that Ct-HBx binds to the promoters of growth-inhibitory miRNAs for transcriptional suppression (Figure 4), presumably through its physical association with transcriptional repressors in the gene promoters [56], [58]. We speculate that this direct transcriptional regulation by Ct-HBx represents one of its major biological functions, since it has been shown to preferentially localize in the nucleus in contrast to the more cytoplasmic-orientated full-length counterpart [4], [44]. In addition, we demonstrated that full-length HBx bound to some miRNA promoters (Figure S2). In some cases full-length HBx bound to similar promoter region with Ct-HBx and in other cases different or even no binding regions. The potential diverse *cis*-regulatory modules (combination of transcription factor binding sites) in these distinct promoter regions and thus transcription factor partners [25] might determine the regulatory function of HBx on miRNA transcription. In addition to miRNAs, our ChIP-chip analysis also demonstrated that Ct-HBx physically associates with promoters of protein-coding tumor-suppressors for gene silencing (Zhu, Cheng *et al.*, unpublished data). Because these down-regulated genes may directly contribute to hepatocarcinogenesis, the molecular basis of such regulations e.g. the identity of specific transcriptional regulators and their functional interaction warrants further investigation.

As HBx is frequently integrated into the host genome in truncated form and over-expressed in HBV-associated HCC [10]–[13], Ct-HBx-mediated transcriptional repression may be one of the reasons underlying the reduced expressions of miR-26a and -29c in HCC cells (Table 2 and Figure S1). One of the limitations of this study is the lack of enough HCC tissue samples for HBx protein expression analysis. In addition, the relatively small case numbers also hinders a solid conclusion to be drawn. Thus, we cannot exclude the possibility of the involvement of other mechanisms such as liver-enriched transcription factors in the transcriptional regulation of these miRNAs in human HCC [59]. We demonstrated that the expression levels of miR-146a and -190, along with miR-26a and -29c, were significantly lower in HCCs compared to the matching non-tumor tissues (Figure 5). Together with the reported down-regulation of miR-30d and -193b in other malignancies [36], [40], [51]–[53], it is conceivable that specific miRNA repression is vital for Ct-HBx-mediated hepatocarcinogenesis. For example, viral-mediated administration of miR-26a in a mouse model of HCC resulted in inhibition of cancer cell proliferation, induction of tumor-specific apoptosis, and dramatic protection from disease progression [31]. We and others have recently demonstrated that miR-26a possessed the tumor-suppressive functions by directly targeting the EZH2 oncogene in cancers e.g. nasopharyngeal carcinoma [30], lymphoma [60] and HBV-associated HCC cells (Figure S3). Moreover, we found that the histone methyltransferase EZH2 [61] was over-expressed in human HCCs and promoted HCC cell growth and tumorigenicity at least partially through activation of Wnt/β-catenin signaling [24], [62]. Therefore, Ct-HBx might promote HCC development via the deregulation of miR-26a control on the EZH2 epigenetic machinery [63]. This notion is further supported by findings from large cohorts of HBV-associated HCCs demonstrating that patients whose tumors had low miR-26a expression had shorter overall survival than those with high tumor miR-26a expression [64].

Another important miRNA transcriptionally repressed by Ct-HBx was miR-29c, whose down-regulation was significantly associated with worse disease-free survival of HCC patients [34]. This miRNA has been shown to repress HCC growth *in vitro* and *in vivo* through promotion of apoptosis by targeting anti-apoptotic molecules, Bcl-2 and Mcl-1 [34]. Apart from apoptosis pathway, we and others have also determined the roles of miR-29 in controlling differentiation, DNA methylation and p53 pathways via repression of YY1 [65], DMNT3A/3B [35] and CDC42 and p85 alpha [66], respectively. Furthermore, miR-146a, -193b and -29c have been shown to inhibit tumor cell invasiveness and metastatic potential by repressing EGFR, urokinase-type plasminogen activator and extracellular matrix proteins, respectively [52], [67], [68]. Taken together, Ct-HBx, via its repressed miRNAs and the corresponding perturbed gene-networks, may not only cause uncontrolled growth placing large numbers of cells susceptible to neoplastic transformation but also promote tumor progression.

In conclusion, this study uncovers the role of HBx, especially the naturally occurring carboxyl-terminal truncated mutant, in regulating cellular miRNAs of human hepatocytes. In contrast to its full-length counterpart, Ct-HBx distinctively down-regulated a set of growth-inhibitory miRNAs and concordantly promoted the growth of hepatocytes. Our integrative miRNA profiling and ChIP-chip analysis also highlights the nuclear *trans*-repressor role of Ct-HBx in miRNA regulation. Together with the miRNA expression analysis in clinical specimens, our findings suggest that Ct-HBx, at least in part, drives the miRNA transcriptional program in HCC development and represents a new therapeutic target for HCC treatment.

## Supporting Information

Figure S1 **Effects of HBx in LO2 immortalized human hepatocyte cell line.** (A) Effect of full-length HBx and Ct-HBx on cell proliferation. Growth of LO2 hepatocytes expressing full-length HBx, HBxΔ35 or empty vector control was determined by cell counting assay. Results are derived from 3 replicates of 2 independent experiments (± SD). (B) Expression of miR-26a and miR-29c in LO2 hepatocytes expressing HBxΔ35 or empty vector control. miRNA expression was measured by quantitative PCR using miScript Reverse Transcription and miScript SYBR Green PCR kits (Qiagen). *, *p*<0.05; **, *p*<0.01; ***, *p*<0.005.
(PPT)

Figure S2 **Binding of full-length HBx and Ct-HBx in miRNA promoters.** ChIP assays were performed with specific HBx antibody in MIHA hepatocytes expressing full-length HBx, HBxΔ35 or EGFP vector control. Coupled with a human promoter microarray, the binding regions of full-length HBx (blue line) and HBxΔ35 (red line) compared to vector control in (A) miR-23a/27a, (B) miR-26a and (C) miR-30d promoters were shown. The Y-axis of the HBx binding maps represents the enrichment ratio (full-length HBx or HBxΔ35/Vector) while the X-axis represents the probe locations relative to the transcription start site (TSS) of miRNAs. Dotted lines indicative of no enrichment are shown as reference. Yellow bars indicate the miRNA promoter amplicon regions in real-time ChIP-PCR assays as shown on the right. The immunoprecipitated DNA corresponding to the miRNA promoters was measured as a percent of input DNA and depicted as relative binding level.
(PPT)

Figure S3 **Effect of ectopic miR-26a over-expression on EZH2 expression in PLC5 HBV associated HCC cell line.** (A) miR-26a expression upon Lipofectamine 2000-mediated transfection of mimics was measured by quantitative PCR using miScript Reverse Transcription and miScript SYBR Green PCR. (B) Western blot analysis of EZH2 expression in PLC5 cells following ectopic expression of miR-26a. β-actin was used as loading control. Signal density was quantified by Glyko BandScan software and defined as the ratio of EZH2 to β-actin. These data suggested that miR-26a post-transcriptionally suppressed EZH2 expression in HCC cells.
(PPT)
