# Supplementary material for: Carboxyl-Terminal Truncated HBx Regulates a Distinct MicroRNA Transcription Program in Hepatocellular Carcinoma Development
Source: PLoS One. 2011 Aug 4;6(8):e22888. doi: 10.1371/journal.pone.0022888 (PMC3150371; doi:10.1371/journal.pone.0022888)
Supplement: Figure S1 — Effects of HBx in LO2 immortalized human hepatocyte cell line. (A) Effect of full-length HBx and Ct-HBx on cell proliferation. Growth of LO2 hepatocytes expressing full-length HBx, HBxΔ35 or empty vector control was determined by cell counting assay. Results are derived from 3 replicates of 2 independent experiments (± SD). (B) Expression of miR-26a and miR-29c in LO2 hepatocytes expressing HBxΔ35 or empty vector control. miRNA expression was measured by quantitative PCR using miScript Reverse Transcription and miScript SYBR Green PCR kits (Qiagen). *, p<0.05; **, p<0.01; ***, p<0.005. (PPT) [file pone.0022888.s001.ppt]

## Slide 1
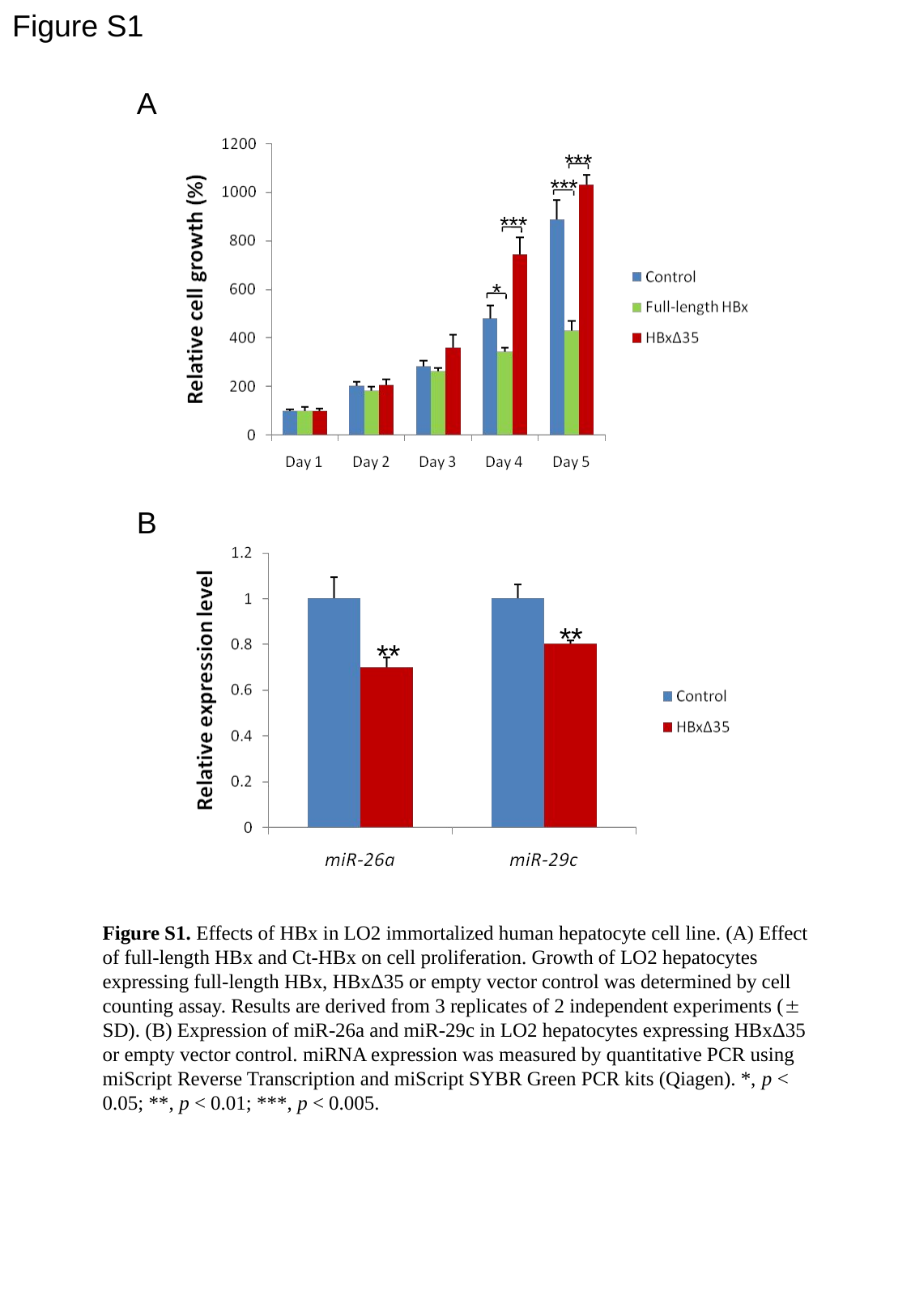

Figure S1
A
***
***
***
*
B
**
**
Figure S1. Effects of HBx in LO2 immortalized human hepatocyte cell line. (A) Effect of full-length HBx and Ct-HBx on cell proliferation. Growth of LO2 hepatocytes expressing full-length HBx, HBxΔ35 or empty vector control was determined by cell counting assay. Results are derived from 3 replicates of 2 independent experiments (± SD). (B) Expression of miR-26a and miR-29c in LO2 hepatocytes expressing HBxΔ35 or empty vector control. miRNA expression was measured by quantitative PCR using miScript Reverse Transcription and miScript SYBR Green PCR kits (Qiagen). *, p < 0.05; **, p < 0.01; ***, p < 0.005.
