# Supplementary material for: Carboxyl-Terminal Truncated HBx Regulates a Distinct MicroRNA Transcription Program in Hepatocellular Carcinoma Development
Source: PLoS One. 2011 Aug 4;6(8):e22888. doi: 10.1371/journal.pone.0022888 (PMC3150371; doi:10.1371/journal.pone.0022888)
Supplement: Figure S2 — Binding of full-length HBx and Ct-HBx in miRNA promoters. ChIP assays were performed with specific HBx antibody in MIHA hepatocytes expressing full-length HBx, HBxΔ35 or EGFP vector control. Coupled with a human promoter microarray, the binding regions of full-length HBx (blue line) and HBxΔ35 (red line) compared to vector control in (A) miR-23a/27a, (B) miR-26a and (C) miR-30d promoters were shown. The Y-axis of the HBx binding maps represents the enrichment ratio (full-length HBx or HBxΔ35/Vector) while the X-axis represents the probe locations relative to the transcription start site (TSS) of miRNAs. Dotted lines indicative of no enrichment are shown as reference. Yellow bars indicate the miRNA promoter amplicon regions in real-time ChIP-PCR assays as shown on the right. The immunoprecipitated DNA corresponding to the miRNA promoters was measured as a percent of input DNA and depicted as relative binding level. (PPT) [file pone.0022888.s002.ppt]

## Slide 1
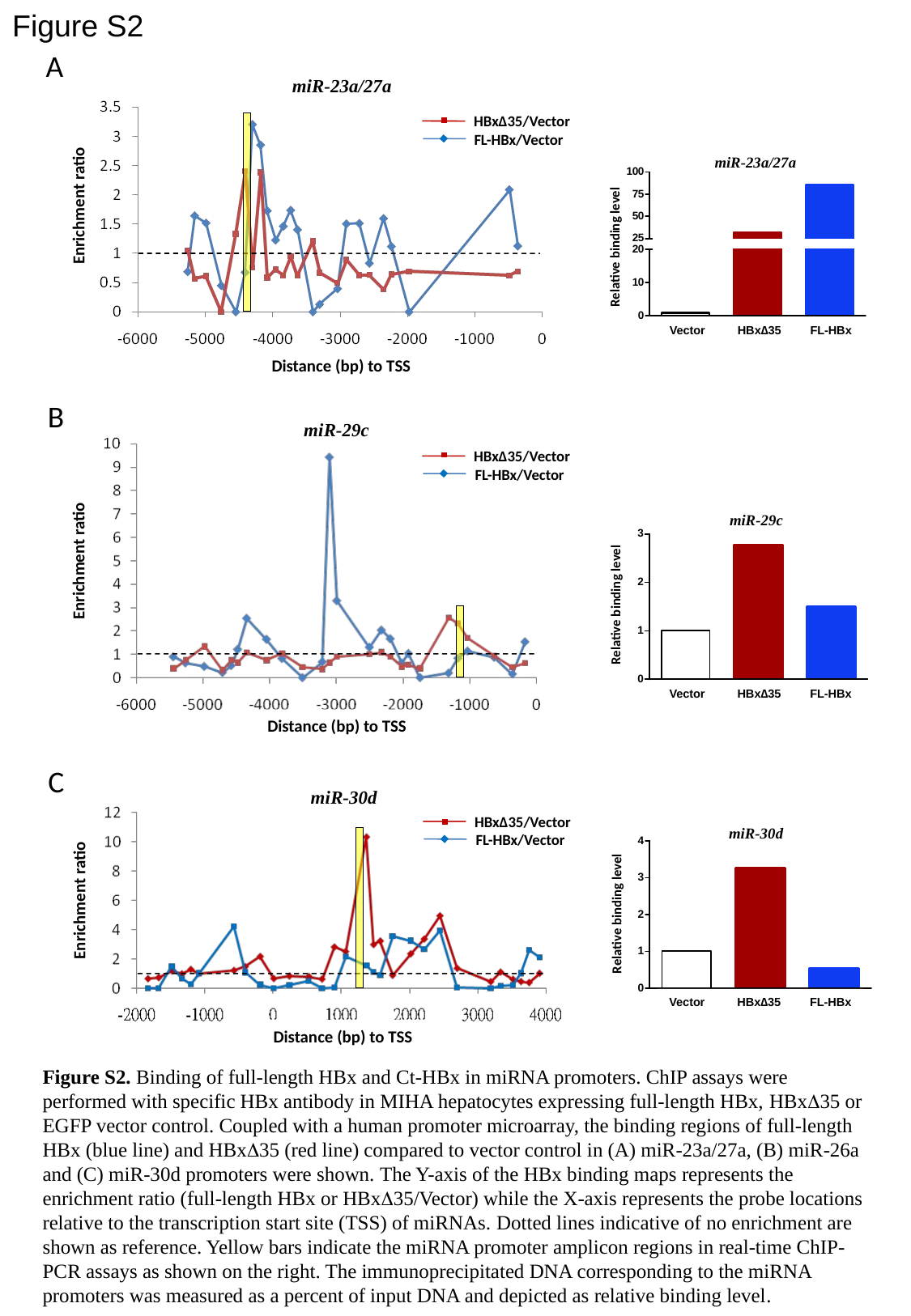

Figure S2
A
miR-23a/27a
HBx∆35/Vector
FL-HBx/Vector
miR-23a/27a
Enrichment ratio
Relative binding level
Vector HBx∆35 FL-HBx
Distance (bp) to TSS
B
miR-29c
HBx∆35/Vector
FL-HBx/Vector
miR-29c
Enrichment ratio
Relative binding level
Vector HBx∆35 FL-HBx
Distance (bp) to TSS
C
miR-30d
HBx∆35/Vector
FL-HBx/Vector
miR-30d
Enrichment ratio
Relative binding level
Vector HBx∆35 FL-HBx
Distance (bp) to TSS
Figure S2. Binding of full-length HBx and Ct-HBx in miRNA promoters. ChIP assays were performed with specific HBx antibody in MIHA hepatocytes expressing full-length HBx, HBxΔ35 or EGFP vector control. Coupled with a human promoter microarray, the binding regions of full-length HBx (blue line) and HBxΔ35 (red line) compared to vector control in (A) miR-23a/27a, (B) miR-26a and (C) miR-30d promoters were shown. The Y-axis of the HBx binding maps represents the enrichment ratio (full-length HBx or HBxΔ35/Vector) while the X-axis represents the probe locations relative to the transcription start site (TSS) of miRNAs. Dotted lines indicative of no enrichment are shown as reference. Yellow bars indicate the miRNA promoter amplicon regions in real-time ChIP-PCR assays as shown on the right. The immunoprecipitated DNA corresponding to the miRNA promoters was measured as a percent of input DNA and depicted as relative binding level.
