# Supplementary material for: Carboxyl-Terminal Truncated HBx Regulates a Distinct MicroRNA Transcription Program in Hepatocellular Carcinoma Development
Source: PLoS One. 2011 Aug 4;6(8):e22888. doi: 10.1371/journal.pone.0022888 (PMC3150371; doi:10.1371/journal.pone.0022888)
Supplement: Figure S3 — Effect of ectopic miR-26a over-expression on EZH2 expression in PLC5 HBV associated HCC cell line. (A) miR-26a expression upon Lipofectamine 2000-mediated transfection of mimics was measured by quantitative PCR using miScript Reverse Transcription and miScript SYBR Green PCR. (B) Western blot analysis of EZH2 expression in PLC5 cells following ectopic expression of miR-26a. β-actin was used as loading control. Signal density was quantified by Glyko BandScan software and defined as the ratio of EZH2 to β-actin. These data suggested that miR-26a post-transcriptionally suppressed EZH2 expression in HCC cells. (PPT) [file pone.0022888.s003.ppt]

## Slide 1
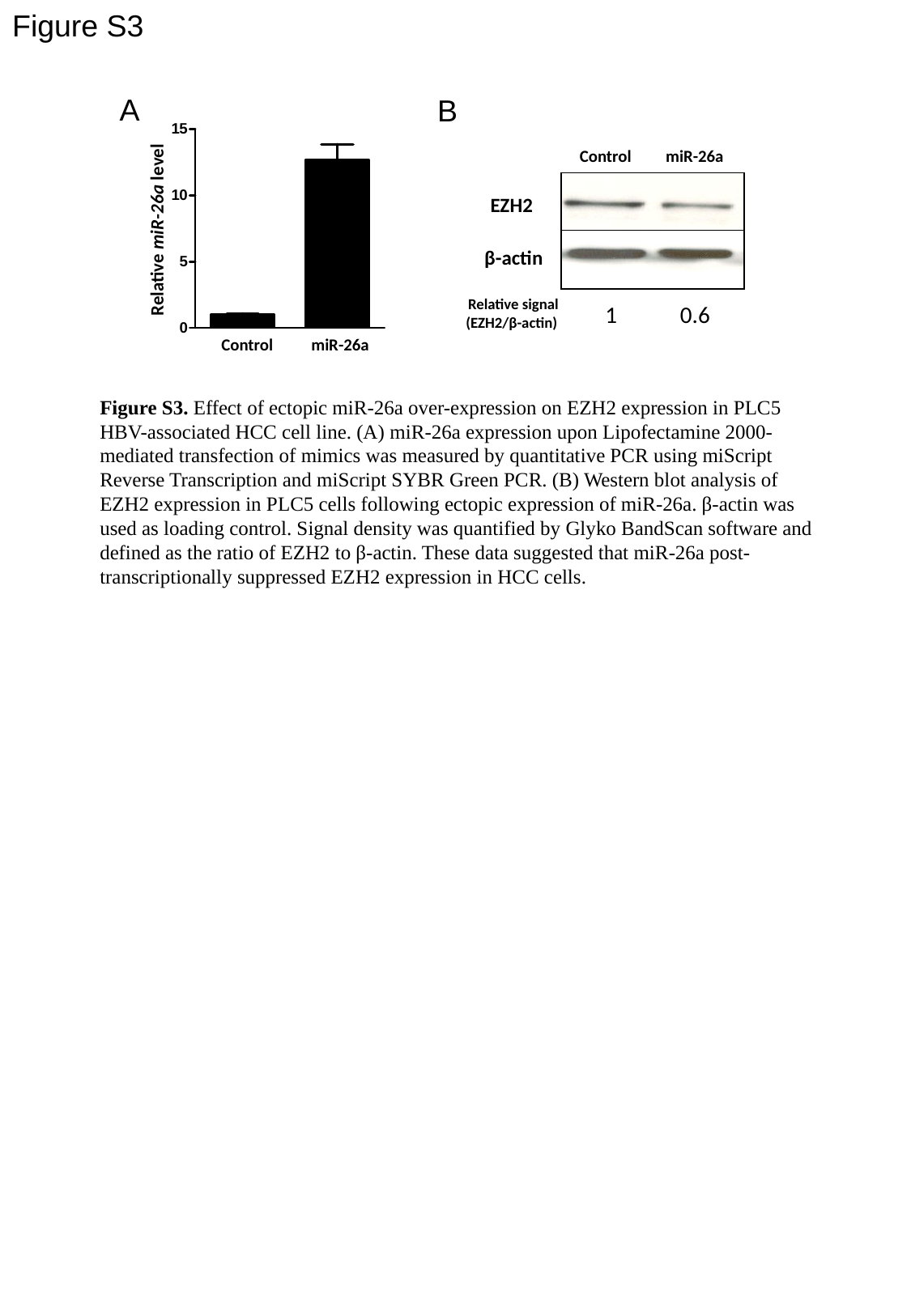

Figure S3
A
B
 Control miR-26a
EZH2
Relative miR-26a level
β-actin
Relative signal
(EZH2/β-actin)
1 0.6
Control miR-26a
Figure S3. Effect of ectopic miR-26a over-expression on EZH2 expression in PLC5 HBV-associated HCC cell line. (A) miR-26a expression upon Lipofectamine 2000-mediated transfection of mimics was measured by quantitative PCR using miScript Reverse Transcription and miScript SYBR Green PCR. (B) Western blot analysis of EZH2 expression in PLC5 cells following ectopic expression of miR-26a. β-actin was used as loading control. Signal density was quantified by Glyko BandScan software and defined as the ratio of EZH2 to β-actin. These data suggested that miR-26a post-transcriptionally suppressed EZH2 expression in HCC cells.
